# Novel Immortalized Human Multipotent Mesenchymal Stromal Cell Line for Studying Hormonal Signaling

**DOI:** 10.3390/ijms25042421

**Published:** 2024-02-19

**Authors:** Alexandra Primak, Natalia Kalinina, Mariya Skryabina, Vladimir Usachev, Vadim Chechekhin, Maksim Vigovskiy, Elizaveta Chechekhina, Nikita Voloshin, Konstantin Kulebyakin, Maria Kulebyakina, Olga Grigorieva, Pyotr Tyurin-Kuzmin, Nataliya Basalova, Anastasia Efimenko, Stalik Dzhauari, Yulia Antropova, Ivan Plyushchii, Zhanna Akopyan, Veronika Sysoeva, Vsevolod Tkachuk, Maxim Karagyaur

**Affiliations:** 1 Faculty of Medicine, Lomonosov Moscow State University, 119234 Moscow, Russiavadimchex97@gmail.com (V.C.); zhanna.fbm@gmail.com (Z.A.);; 2 Institute for Regenerative Medicine, Medical Research and Education Center, Lomonosov Moscow State University, 119234 Moscow, Russia

**Keywords:** multipotent mesenchymal stromal cells (MSCs), immortalization, telomerase reverse transcriptase (TERT), hormone sensitivity, differentiation, proliferative potential, ASC52telo

## Abstract

Multipotent mesenchymal stromal cells (MSCs) integrate hormone and neuromediator signaling to coordinate tissue homeostasis, tissue renewal and regeneration. To facilitate the investigation of MSC biology, stable immortalized cell lines are created (e.g., commercially available ASC52telo). However, the ASC52telo cell line has an impaired adipogenic ability and a depressed response to hormones, including 5-HT, GABA, glutamate, noradrenaline, PTH and insulin compared to primary cells. This markedly reduces the potential of the ASC52telo cell line in studying the mechanisms of hormonal control of MSC’s physiology. Here, we have established a novel immortalized culture of adipose tissue-derived MSCs via forced telomerase expression after lentiviral transduction. These immortalized cell cultures demonstrate high proliferative potential (up to 40 passages), delayed senescence, as well as preserved primary culture-like functional activity (sensitivity to hormones, ability to hormonal sensitization and differentiation) and immunophenotype up to 17–26 passages. Meanwhile, primary adipose tissue-derived MSCs usually irreversibly lose their properties by 8–10 passages. Observed characteristics of reported immortalized human MSC cultures make them a feasible model for studying molecular mechanisms, which regulate the functional activities of these cells, especially when primary cultures or commercially available cell lines are not appropriate.

## 1. Introduction

Hormones and neuromediators are important “global” regulators of regeneration [1], and multipotent mesenchymal stromal cells (MSCs) are primary targets for these molecules. These cells express a wide range of membrane receptors for various hormones and neuromediators. However, the investigation of molecular mechanisms of hormonal signaling in MSCs is often limited by a short lifetime of cultured primary MSCs (pMSCs) due to low telomerase activity and proliferation as well as rapid replicative senescence [2]. Moreover, genetic knock-in/out of signaling molecules by conventional genome editing (to elucidate their function in a certain signaling cascade) often requires clone selection or expansion of the edited cell population, and therefore, the usage of a genome-editing approach is restrained in primary cells [3]. Furthermore, primary MSCs demonstrate a remarkable heterogeneity in hormonal responses, which also interferes with obtaining definitive results. MSC cultures with an expanded proliferative potential able to circumvent these limitations would be a convenient tool for establishing the role of individual signaling molecules and cascades in tissue renewal and regeneration.

In this study, we created immortalized MSC cultures (iMSCs) via forced telomerase reverse transcriptase overexpression after lentiviral transduction. Telomerase overexpression allows us to increase telomere length and prolong cell culture proliferation. At the same time, according to the literature data, telomerase expression does not affect the properties of primary cell cultures and has the least effect on genome stability compared to other immortalization approaches, involving the suppression of p16 or p53 [4,5].

Obtained iMSC cultures retain primary MSC-specific immunophenotypes, differentiation potential and sensitivity to various hormones and neuromediators up to 17–26 passages. Compared to iMSCs, the commercially available MSC line ASC52telo demonstrated an aberrant response to several hormones and impaired adipogenic differentiation potential. Developed iMSC cultures could be further used in a wide range of research projects, including those which aim to elucidate the hormonal regulation of MSC functioning.

## 2. Results

### 2.1. Immortalized MSCs (iMSCs) Demonstrate Higher Telomerase Activity and Proliferation Potential Together with Delayed Replicative Senescence

**Figure 1:**
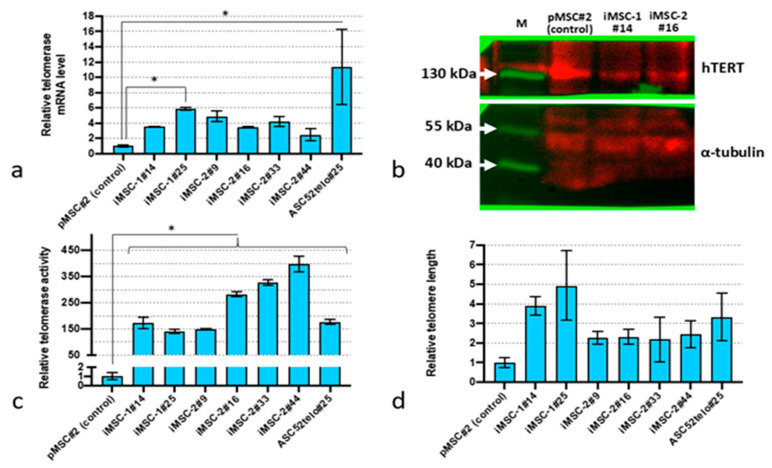
Telomerase expression in immortalized MSCs. (**a**) TERT mRNA expression level before (pMSCs) and after the immortalization procedure (iMSC-1 and iMSC-2) established using qPCR; (**b**) Western blot analysis of TERT protein content; (**c**) telomerase activity assessment by qPCR assay; (**d**) relative telomere length. *—*p* < 0.05 (ANOVA multiple pairwise comparison, Tukey test), n = 3. Data are presented as mean ± standard deviation.

Primary MSC (pMSC) transduction with lentiviruses encoding human telomerase cDNA caused a sustained increase in telomerase expression (Figure 1a). The level of TERT mRNA in such MSC cultures (termed iMSC-1 and iMSC-2 for “immortalized”) was 2–5-fold higher compared to pMSCs (*p* < 0.05, n = 3), but lower compared to ASC52telo cells. Moreover, we did not observe significant changes in telomerase mRNA expression in iMSC cultures with passaging (up to the passage 44). Western blot analysis did reveal any elevation in telomerase protein content in iMSC-1 and iMSC-2 cultures compared to pMSCs (Figure 1b). However, the telomerase activity and telomere length were significantly higher in iMSC-1 and iMSC-2 compared to pMSCs (*p* < 0.05, n = 3) (Figure 1c,d).

**Figure 2:**
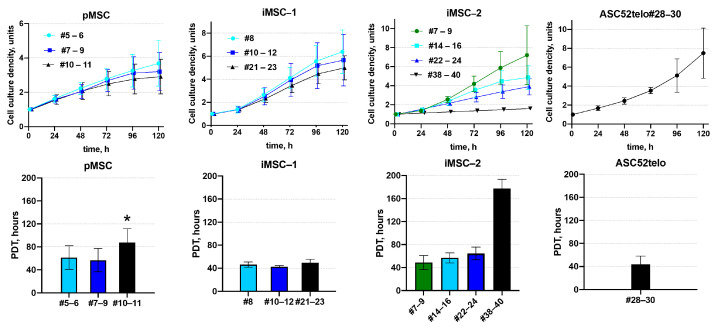
iMSC proliferation. Proliferation of pMSC and iMSC cultures was assessed via measuring the dynamics of cell density (upper panels) and calculating the population doubling time (PDT, lower panels). #N—passage number, *—*p* < 0.05—pMSCs PDT vs. iMSCs PDT at the corresponding passage, n ≥ 4. Data are presented as mean ± standard deviation.

Consistent with high telomerase activity, iMSC-1 and iMSC-2 demonstrated higher proliferation rates compared to pMSCs. Thus, in pMSC cultures, the proliferation rate decreased 1.4-fold between the 5th and 11th passages with the population doubling time increasing from 61.5 ± 20.6 to 87.6 ± 24.0 h. At the same time, the iMSC proliferation rate did not significantly change between the 5th and 11th passages with an average doubling time of 57.6 ± 12.1 h. The population doubling time in iMSCs at the 11th passage was 1.7-fold shorter compared to pMSCs at the same passage: 50.5 ± 9.8 h and 87.6 ± 24.0 h, respectively (*p* < 0.05, n ≥ 6). The iMSC proliferation rate decreased after 30 passages with the 2-fold increase in population doubling time between the 24th and 38–44th passages: from 59.1 ± 11.7 h to 117.8 ± 15.8 h, respectively (*p* < 0.05, n = 3) (Figure 2).

In contrast to iMSCs, the ASC52telo cell line retained a high proliferation rate even at the 30th passage. At the same time, ASC52telo cells demonstrated lower contact inhibition, compared to pMSCs and iMSC. They continue to proliferate without any apparent slowing down even when the monolayer is formed, whereas pMSC and iMSC cells slow down proliferation in confluent cultures.

**Figure 3:**
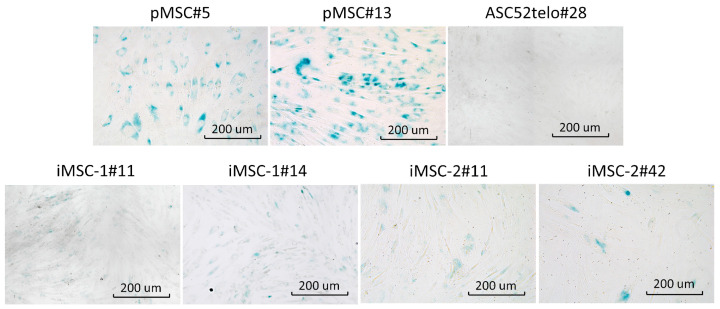
iMSCs senescence. Beta-galactosidase activity (blue color) was analyzed in pMSCs and ASC52telo cell line (upper panel’s row) as well as iMSCs-1 and iMSCs-2 cell lines. Passage number is indicated for each cell line.

iMSCs also demonstrated delayed replicative senescence compared to pMSCs as revealed by cell culture beta-galactosidase staining, specific to senescent cells (Figure 3). However, at the passage 42 beta-galactosidase positive cells appeared in iMSC-2 culture to mark its aging. Commercially available cell line ASC52telo demonstrated no signs of senescent cells at passage 28.

### 2.2. Immortalized MSCs (iMSCs) Retain MSC-Specific Immunophenotype and Differentiation Potential

**Figure 4:**
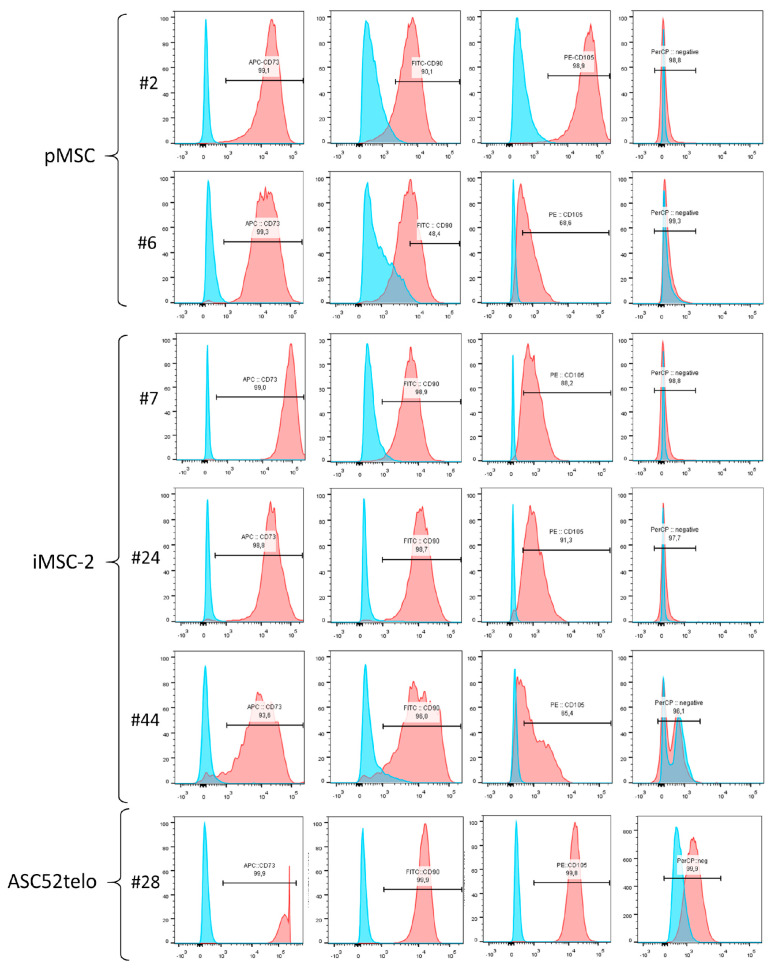
Flow cytometry analysis of MSC-specific markers in pMSC, iMSC and ASC52telo cell lines. #N—passage number.

Primary cells, which were used for immortalization, demonstrated the CD73+/CD90+/CD105+/CD14−/CD19−/CD20−/CD34−/CD45−/HLA-DR immunophenotype suggested for MSC characterization by the International Society for Cell Therapy (ISCT) [6,7] (Figure 4, pMSC#2).

Immortalization of pMSCs at early passages (first or second) did not affect their immunophenotype profile (Figure 4, compare pMSC#2 and iMSC-2#7). Furthermore, iMSCs retain MSC-specific immunophenotype for many passages and only at the 44th passage a decline in marker antigen expression by iMSCs was observed. Thus, the number of CD73+ cells decreased from 99.2% to 93.6%, CD90+ cells from 99.1% to 96%, and CD105+ cells from 96.1% to 65.4%, between the 7th and 44th passages (Figure 4, iMSC-2#44). Of note, the ASC52telo cell line also demonstrated “classic” MSC immunophenotype and retained it at the 28th passage.

**Figure 5:**
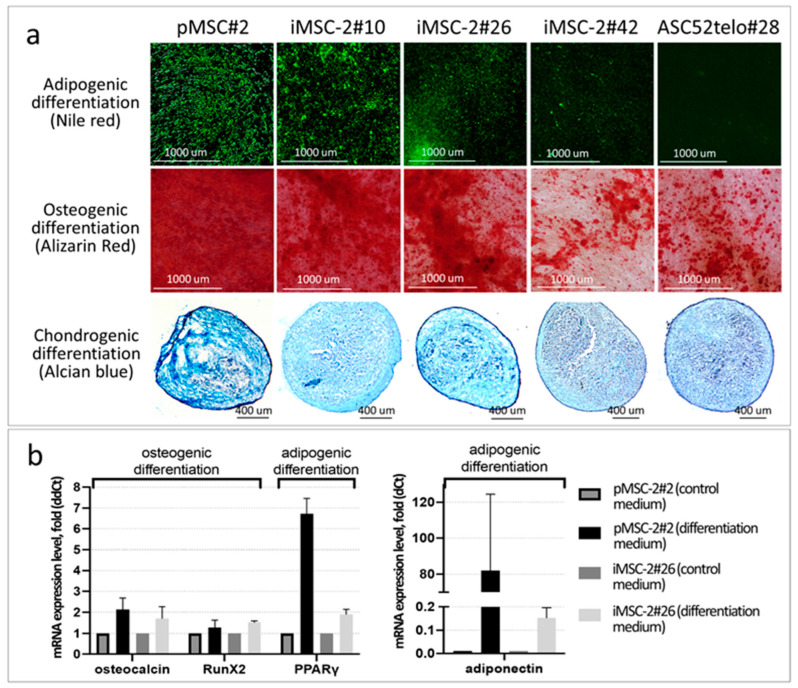
iMSC-2 differentiation potential. (**a**) MSCs cultured in appropriate differentiation medium for 2 weeks. Adipogenic differentiation was assessed by Nile red staining of accumulated lipids (green fluorescence), osteogenic differentiation—by Alizarin red staining of calcium deposits (red color) and chondrogenic differentiation—by Alcian blue staining of cartilage acidic glycosaminoglycans (light blue color); (**b**) relative expression level of marker genes of osteogenic (osteocalcin, Runx) and adipogenic (PPARgamma, adiponectin) differentiation in pMSCs and iMSCs cultured for 15 days in ordinary complete growth medium or appropriate differentiation medium.

The immortalization procedure also did not affect MSCs’ differentiation potential. iMSCs readily differentiated into adipocytes, osteocytes and chondrocytes in appropriate differentiation medium at early (#10–14) passages (Figure 5 and Figure S1). However, their differentiation potential gradually decreased up to passages #21–42 (Figure 5 and Figure S1), which is one of the hallmarks of cell culture senescence. Direct comparison of differentiation potential of iMSCs and ASC52telo cells has revealed that the latter exhibit an impaired adipogenic potential (at the appropriate passages), which is consistent with previous observations [8] (Figure 5).

### 2.3. Hormonal Sensitivity of iMSCs Does Not Differ from Primary MSCs

**Figure 6:**
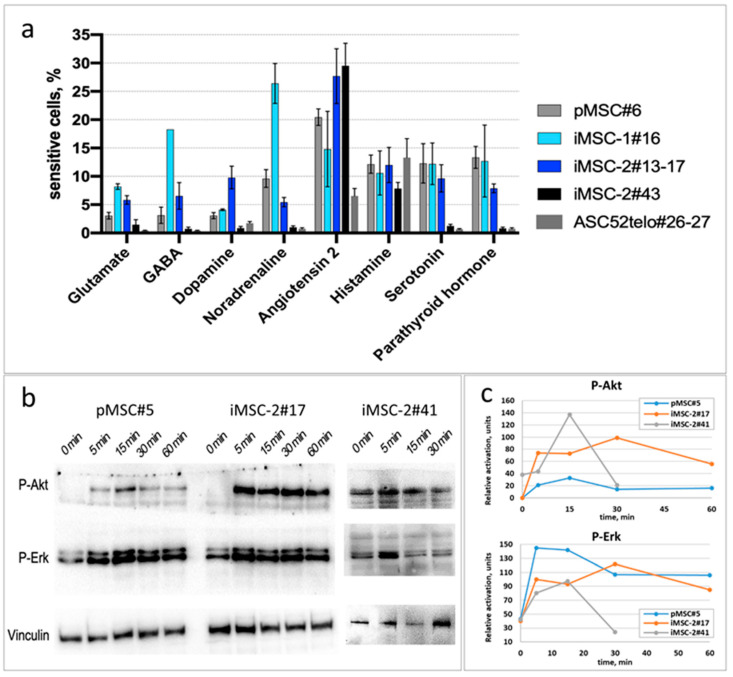
iMSC response to hormones. (**a**) The portion of cells, responded to glutamate (10^−5^ M), GABA (2 × 10^−5^ M), dopamine (10^−5^ M), noradrenaline (10^−6^ M), angiotensin II (10^−8^ M), histamine (10^−6^ M), 5-HT (10^−5^ M) and PTH (10^−8^ M) by increase in cytoplasmic Ca^2+^ influx. (**b**,**c**) AKT and ERK phosphorylation in response to insulin. Western blot of MSC samples with P-AKT and P-ERK specific antibodies. (**c**) Vinculin content was used as a reference for densitometry of P-AKT and P-ERK bands.

Immortalization may significantly alter MSC sensitivity to various hormones, as we demonstrated earlier for the ASC52telo cell line [8]. Here, we have demonstrated that MSC immortalization via hTERT-encoding lentivirus transduction does not affect their hormonal sensitivity. Thus, iMSCs responded to glutamate, GABA, dopamine, noradrenaline, angiotensin II, histamine, 5-HT and parathormone by an increase in cytoplasmic Ca^2+^ influx. The percentage of responding cells in iMSC cultures corresponded to that in primary MSCs (Figure 6a). In contrast to iMSCs and pMSCs, the ASC52telo cell line demonstrated an impaired sensitivity to the majority of hormones tested, including glutamate, GABA, dopamine, noradrenaline, 5-HT and parathormone (*p* < 0.05, n ≥ 4). The sensitivity profile of primary MSC cultures at different passages to hormones is shown in Figure S2.

Furthermore, an important peculiarity of pMSC hormonal response is the ability to increase the sensitivity to noradrenaline after the treatment by noradrenaline or 5-HT [9]. We have previously shown that hormonal sensitization is significantly reduced in the ASC52telo cell line compared to primary MSCs [10]. In contrast, iMSCs-2 demonstrated an increased sensitivity to noradrenaline after the treatment by noradrenaline just as primary MSCs do (Figure S3).

Since insulin is an important regulator of adipose tissue-derived MSCs, we assessed the insulin sensitivity of iMSCs. Similarly to pMSC, iMSCs (up to the 41st passage) respond to insulin by the activation of PI3K/Akt and Ras/ERK signaling pathways. The dynamic of AKT phosphorylation was similar in pMSCs and iMSCs (up to the 41st passage); however, in iMSC-2#41, we observed an elevated baseline p-AKT level (Figure 6b,c), common in senescence cell cultures. In contrast to those, ASC52telo cells exhibited persistent elevated baseline p-AKT and p-ERK levels without any apparent increase in response to insulin, as was previously published [8].

## 3. Discussion

Infinite cell lines are an irreplaceable tool for many biological assays [11,12,13]. Therefore, several approaches were developed to convert primary cells, isolated from different tissues, into long-lived cultures with consistent biological functions [14,15,16]. In this study we created an immortalized adipose tissue-derived MSC culture via ectopic expression of the human TERT gene, similar to what had been previously described for bone marrow stromal cells [17].

In previous reports, the ectopic expression of hTERT improved telomerase activity, stabilized telomere length, slowed down cell senescence, and prolonged the lifespan of culture [18,19]. Consistent with these reports, our iMSC cultures also had a greater proliferation potential than primary MSCs as well as delayed replicative senescence. Immortalized MSCs lines obtained in this study demonstrated normal contact inhibition, just as was shown for bone marrow-derived mesenchymal cells [20], whereas ASC52telo cells did not slow down their proliferation even in the dense cultures. At the same time, the longevity of obtained iMSC cell lines was finite, since these cells slowed down their proliferation with time. Although hTERT expression and activity as well as the length of telomeres persisted for all passages, iMSCs demonstrated senescence signs (increased PDT and beta-galactosidase activity) after 30 passages. Presumably, other mechanisms of cell senescence (e.g., p16/p53-p21-induced or mitochondrial dysfunction-associated senescence) are involved in the aging of cells stably expressing hTERT [21]. Our data are consistent with previously published studies describing TERT-immortalized bone marrow-derived MSCs [17]. These data indicate that ectopic hTERT expression per se is not sufficient for adipose tissue-derived MSCs immortalization, and postpones rather than prevents culture exhaustion. Additional spontaneous mutations in protooncogenes or tumor suppressor genes could lead to the tumorigenic transformation of cells, making them immortal, with unlimited proliferation [22].

Immortalized cells obtained in this study also retained the MSC-specific immunophenotype and readily differentiated towards adipocytes, chondrocytes and osteocytes in appropriate differentiation medium. These data contrast with results of previous study, where compromised differentiation abilities of ASC52telo cells were reported. Thus, ASC52telo cells have impaired adipogenic potential, which is consistent with previous observations [8].

The most advantageous feature of iMSCs is their ability to respond to hormones and neuromediators in a fashion similar to primary MSCs. These data indicate that the immortalization procedure via lentivirus-mediated ectopic hTERT expression does not interfere with MSC physiology. These iMSC properties make them a stable and convenient object for further fundamental and applied research, i.e., for testing hypotheses of MSC involvement in tissue renewal and regeneration as well as in the pathogenesis of metabolic and vascular diseases [23,24]. This is particularly important since some immortalized MSC lines (e.g., commercially available adipose tissue-derived ASC52telo cell line) are not suitable for such studies, because of their impaired sensitivity to hormones: insulin [8], noradrenaline [10], serotonin, GABA and glutamate, and compromised adipogenic potential [8,25].

The expanded proliferative potential of iMSCs, together with the preservation of the properties of the original cell culture, makes iMSCs a convenient object for genomic editing, including for the purpose of CRISPR/Cas9-mediated knock-in genome modifications. Such modifications in primary cell cultures are hampered by the impossibility of obtaining genetically modified cell clones due to the low proliferative potential of primary cultures and low efficiency of CRISPR/Cas9-mediated knock-in in such cultures [26,27,28].

A number of studies demonstrate that immortalized cell cultures (within one cell type) may differ significantly in their properties both from each other and from the starting primary cells [8,29,30]. The reasons for this remain to be established. Presumably, it may be due to both individual donor characteristics and the influence of procedures of lentiviral transduction and genetic modification (or immortalization) [23,31,32]. How significant the contribution is of each of these factors to the properties of the obtained immortalized adipose tissue-derived MSC culture remains to be elucidated on a larger number of primary MSC cultures with direct comparison of their properties before and after modification.

## 4. Materials and Methods

### 4.1. Cell Cultures

Primary multipotent mesenchymal stromal cells isolated from human subcutaneous fat were obtained from the biobank of the Institute for Regenerative Medicine, Lomonosov Moscow State University (https://human.depo.msu.ru (accessed on 13 February 2024)). They served as ancestor cell cultures for immortalization and obtaining immortalized cells (iMSCs), as well as a reference during the characterization experiments. ASC52telo, a commercially available immortalized human adipose tissue-derived MSC cell line, was obtained from ATCC (Manassas, VA, USA, #SCRC-4000).

MSCs were cultured in Advance Stem Cell Basal Medium (HyClone, South Logan, UT, USA, #SH30879.02) containing 10% Advance Stem Cell Growth Supplement (HyClone, #SH30878.01) and 1× antibiotic/antimycotic mixture (Gibco, Grand Island, NY, USA, #15240062). All experiments with primary isolated MSCs were performed up to passage 8 and with immortalized MSCs up to passage 44. All procedures with patient tissue samples were performed in accordance with the Declaration of Helsinki and approved by the Ethical Committee of Lomonosov Moscow State University (#IRB00010587), protocol #4 (2018).

The HEK293T line (ATCC, #CRL-3216™) was used for lentiviral particle (LVP) assembly. HEK293T was cultured on Dulbecco’s Modified Eagle Medium (DMEM) with high glucose (Gibco, #11965092) containing 10% fetal bovine serum (FBS) (ThermoFisher Scientific, Waltham, MA, USA, #26140079), 1× antibiotic/antimycotic mixture (Gibco, #15240062) and 1× L-Gln (Gibco, #11539876). LVP production was performed in a similar culture medium containing 2% inactivated fetal bovine serum (FBS) (ThermoFisher Scientific, #26140079). All cell cultures were cultured at 37 °C and 5% CO_2_ in a Binder CB150 incubator (BINDER GmbH, Tuttlingen, Germany). The culture medium was changed every 3–4 days.

### 4.2. hTERT-Encoding Lentivirus Construction

To obtain a transfer plasmid, hTERT cDNA was cloned into pVLT plasmid (Eurogen, Moscow, Russia) using EcoRI and SalI restriction sites followed by cloning puromycin-resistant expression cassette (puroR) using SalI and KpnI sites (Figure S4 for resulting pVLT-EF1a-hTERT-puroR map). hTERT cDNA was amplified from reverse-transcribed total human mRNA using the following primers: 5′-CCACCGAATTCGCCACCATGCCGCGCGCTCCCCGCTGCCGAGCCGTGCGCT-3′ and 5′-GCGTCGTCGACTCAGTCCAGGATGGTCTTGAAGTCTGAGGGCAGTGCCGGGTTG-3′ and Magnus reverse transcriptase (Evrogen, Moscow, Russia, #SK006S).

To achieve lentivirus production, HEK293T cells were transfected with pVLT-EF1a-hTERT-puroR together with lentiviral packaging plasmids using PEI transfection protocol [33]. Viral supernatant was collected 64–72 h after transfection, filtered through a 0.45 μm filter and applied on primary MSCs (MOI ~20). To select transduced MSCs, 10 days after viral transduction, cells were cultured in the presence of 1 μg/mL of puromycin for the next 10–14 days. MSCs that did not receive puroR cassette and therefore did not express puromycin N-acetyltransferase died within 3–5 days of incubation with puromycin. Several iMSC cultures (iMSC-1/2/3) were obtained, each from an individual donor.

### 4.3. Human TERT Expression and Activity

Human TERT gene expression in iMSCs was confirmed by qPCR and immunoblotting. Direct-zol RNA Miniprep kit (Zymo Research, Irvine, CA, USA, #R2052) was used to isolate total RNA from cell lysates (including the DNAase treatment step). Reverse transcription was performed using the MMLV RT kit (Evrogen, Russia, #SK021), and for qPCR, the 5X qPCRmix-HS SYBR kit (Evrogen, Russia, #PK147L) was used. Human *TERT* mRNA was detected using primers and amplification parameters listed in Table S1. The signal obtained was normalized to the expression level of *RPLP0* (housekeeping gene)—for details, see Table S1.

For immunoblotting, pMSC and iMSC lysates were separated in 12% polyacrylamide gel according to a standard protocol followed by protein transfer to a PVDF membrane. Human telomerase was detected using anti-telomerase reverse transcriptase antibody [2C4] (Abcam, Waltham, MA, USA, #ab5181) with the subsequent normalization to alpha tubulin (TUBA1A) content using the alpha tubulin antibody (B-5-1-2) (Santa Cruz Biotechnology, Inc., Dallas, TX, USA, #sc-23948). Horseradish peroxidase-labeled rabbit anti-mouse antibodies P-RAM Iss (Imtek, Moscow, Russia) were used as secondary antibodies. PageRuler™ Prestained Protein Ladder, 10 to 180 kDa (ThermoFisher Scientific, #26616), was used as a molecular weight marker. The PVDF membrane was stained using Clarity™ Western ECL Substrate kit (Bio-Rad, Hercules, CA, USA, #1705060), and signal registration was performed using the Bio Rad ChemiDoc MP Imaging System (Bio-Rad, USA).

The functional activity of the human TERT gene was confirmed using a Telomerase Activity Quantification qPCR Assay Kit (ScienCell Research Laboratories, Carlsbad, CA, USA, #8928) and Relative Human Telomere Length Quantification qPCR Assay Kit (ScienCell Research Laboratories, #8908) according to the manufacturer’s recommendations. pMSCs and ASC52telo were used as references here and following characterization experiments.

### 4.4. MSC Immunophenotyping

pMSC, iMSC and ASC52telo cell immunophenotypes were analyzed using an anti-human MSC Phenotyping Cocktail Kit, REAfinity (Miltenyi Biotec, Bergisch Gladbach, Germany, #130-125-285). Cells were detached from culture dishes, stained according to the manufacturer’s protocol and analyzed on BD FACS Aria III (BD, Franklin Lakes, NJ, USA).

### 4.5. MSC Proliferation

To assess proliferation potential, pMSCs and iMSCs were cultured until they reached a radical slowdown of proliferative activity (replicative senescence)—for iMSC cultures up to ~35–40 passages. Each passaging was performed at a 1:3 ratio, and cell samples were cryopreserved at different passages.

To assess the cell culture doubling rate at different passages, pMSCs, iMSCs and ASC52telo cells were seeded into 6-well plates (100,000 cells per well) followed by automated picture registration using the IncuCyte^®^ ZOOM Live Cell Analysis System (EssenBioscience, Ann Arbor, MI, USA) for 120 h (16 fields of view per 1 well). The acquired images were analyzed using the embedded software by applying a “mask” and calculating the percentage of cell confluent that directly correlates with cell proliferation.

### 4.6. MSC Hormonal Sensitivity

The sensitivity of iMSC cell cultures to hormones was evaluated by their ability to respond to noradrenaline, 5-hydroxytryptamine, glutamate, γ-aminobutyric acid, dopamine, parathyroid hormone, angiotensin II or histamine, with the activation of the Gq/11-phospholipase-C signaling cascade, as well as by the activation of Ras/Erk and PI3K/Akt signaling cascades in response to insulin.

Activation of G-protein-coupled receptors was assessed using Ca^2+^ imaging after treatment with either noradrenaline (1 µM, Abcam, Cambrige, UK, #ab120717), 5-HT (10 µM, Abcam, Cambridge, UK, #ab120528), glutamate (1 µM, Abcam, Cambridge, UK, #ab120049), γ-aminobutyric acid (1 µM, Abcam, Cambridge, UK, #ab120359), dopamine (50 µM, Sigma-Aldrich, Burlington, MA, USA, #H8502), parathyroid hormone (10 nM), angiotensin II (5 nM, Abcam, Cambridge, UK, #ab120183) or histamine (1 µM, #H7125). Cells were grown at low density to prevent cell-to-cell communication during calcium imaging. The cells were loaded with Fluo-8 (4 µM, Abcam, Cambrige, UK, #ab142773) in Hanks’ solution with 20 mM Hepes for 1 h before the experiment. To measure the percentage of the responded cells, we recorded the baseline for 5 min, then added noradrenaline once. Ca^2+^ transients were measured in individual cells using an inverted fluorescent microscope Nikon Eclipse Ti equipped with an objective CFI Plan Fluor DLL 10×/0.3 (Nikon, Tokyo, Japan) and with a digital EMCCD camera Andor iXon 897 (Andor Technology, Belfast, UK) and Nikon Eclipse Ti2 equipped with an objective CFI Plan Fluor DL 10XF CH, NA0.3 (Nikon, Tokyo, Japan) and with a digital sCMOS camera Photometrics Kinetix (Teledyne Photometrics, Tucson, AZ, USA). We used simultaneous measuring of 4 × 4 fields of view in the large image mode to increase the number of analyzed cells. Movies were analyzed using NIS-Elements AR 5.42.02 (Nikon) and ImageJ 1.53v software. Alterations of cytosolic Ca^2+^ were quantified by relative changes in the intensity of Fluo-8 fluorescence (ΔF/F0) in individual cells. The percentage of responded cells was measured as the ratio of the number of responded cells to the number of all the analyzed cells.

To assess iMSCs’ (at passage 17 and 41) and pMSCs’ (passage 5) sensitivity to insulin, the cells were seeded into 6-well plates at 30% confluent and cultured for 24 h. Before insulin stimulation, cells were incubated in serum-free low-glucose DMEM medium for 24 h. To trigger insulin signaling (activation of Ras/Erk and PI3K/Akt cascades), 100 nM insulin (Sigma-Aldrich, Burlington, MA, USA, #I3536) was added to iMSC and pMSC cultures. At 5, 15, 30 and 60 min after insulin application, cells were lysed in sample buffer (62.5 mM Tris-HCl pH 6.8, 2.5% SDS, 0.002% Bromophenol Blue, 5% β-mercaptoethanol, 10% glycerol), and samples were preserved and further analyzed using Western blot as was previously described [8]. Cell lysates were separated in polyacrylamide gel and transferred to a PVDF membrane, which was further stained with antibodies to activated (phosphorylated) forms of Erk1/2 and Akt: Phospho-p44/42 MAPK (Erk1/2) (Thr202/Tyr204) (Cell Signaling Technology, Danvers, MA, USA, #9106), Phospho-Akt (Thr308) (Cell Signaling Technology, #4056) and Vinculin (Sigma-Aldrich, #V4139) as a housekeeping protein. Then, the membrane was washed and stained with anti-rabbit (Sigma, #DC03L) or anti-mouse (Sigma, #DC02L) secondary antibodies, conjugated with horseradish peroxidase for 1 h. The signal was registered using Bio Rad ChemiDoc MP Imaging System (Bio-Rad, USA).

### 4.7. MCS Multilineage Differentiation Assays

To assess the differentiation potential, iMSCs, pMSCs and ASC52telo were differentiated toward osteogenic, chondrogenic and adipogenic lineages in vitro, and their ability to express specific lineage mRNA markers (PPARγ and adiponectin for adipogenic differentiation, osteocalcin and RunX2 for osteogenic differentiation) was assessed.

#### 4.7.1. Osteogenic Differentiation

To assess the osteogenic potential, iMSCs, pMSCs and ASC52telo were cultured in 12-well plates in complete growth medium for 15 days until the dense monolayer was achieved. Osteogenic differentiation was induced using a Mesenchymal Stem Cell Osteogenesis Kit (Merck, Darmstadt, Germany, #SCR028) according to the manufacturer’s instructions. The medium for osteogenic differentiation was based on DMEM low-glucose medium (Gibco, #11885084) supplemented with 10% of FBS, CaCl2 (100 mg/L), dexamethasone (0.1 μM), ascorbic acid (0.2 mM), glycerol-2-phosphate (10 mM), and L-glutamine (2 mM). Control cells were cultured in DMEM low glucose + 10% FBS medium. The medium was replaced every two days. On day 15, the medium was removed, and cells were fixed in 70% ethanol for 1 h. Cells were washed with water and stained with Alizarin red (Sigma, #A5533-25G). The images of the stained cultures were captured with a Nikon Eclipse Ti2 microscope equipped with a Kinetix camera (Teledyne Photometrics, Tucson, AZ, USA) and analyzed using NIS Elements AR 5.40.02 software. The effectiveness of MSC osteogenic potential was assessed by the staining intensity of mineral deposits, primarily, calcium carbonate in the extracellular matrix.

#### 4.7.2. Adipogenic Differentiation

To assess the adipogenic potential, iMSCs, pMSCs and ASC52telo were cultured in 12-well plates in complete growth medium for 15 days until the monolayer was achieved. Adipogenic differentiation was induced by replacing the growth medium with DMEM low glucose (Gibco, #11885084) supplemented with 10% of FBS, insulin (10 μg/mL), IBMX (0.5 mM) and dexamethasone (1 μM), according to the previously described protocol [8]. Control cells were cultured in DMEM low glucose + 10% FBS medium. The medium was replaced every two days. On day 21, cells were washed with HBSS (Hanks’ Balanced Salt Solution) (PanEko, Moscow, Russia, #P020n) supplemented with 20 nM of HEPES (Dia-M, Moscow, Russia, #3350.0100) and then incubated in HBSS with 20 nM HEPES and 1 mM Nile red for 1 h in a cell culture incubator at 37 °C. The images of the stained cultures were captured with a Nikon Eclipse Ti2 microscope equipped with a Kinetix camera (Teledyne Photometrics) and analyzed using NIS Elements AR 5.40.02 software. The effectiveness of MSC adipogenic differentiation was evaluated by the staining intensity of cells with lipophilic dye Nile red (Sigma-Aldrich, #19123).

#### 4.7.3. Chondrogenic Differentiation

Chondrogenic differentiation of pMSCs and iMSCs was performed using a StemPro^®^ Chondrogenesis Differentiation Kit (Gibco, #A1007101) according to the manufacturer’s recommendations. Briefly, 5 μL drops of MSCs suspension containing 1.6 × 10^7^ viable cells/mL were applied to the 24-well plates and incubated for 2 h at 37 °C in a cell incubator under high humidity. Then, the differentiation medium was added and replaced every 2–3 days. Spheroids obtained after 14 days of differentiation were frozen in Tissue-Tek O.C.T. Compound (Sakura Finetek USA, Inc., Torrance, CA, USA, #4583), and 10 μm thick sections were prepared using the Leica CM1850 cryotome (Leica, Wetzlar, Germany). The cryosections obtained were fixed in buffered 10% formalin (Panreac, Barcelona, Spain, #131328), stained with a chondrospecific dye—0.1% alcian blue solution, pH 4.5 (Sigma-Aldrich, #6A3217)—then embedded under coverslips. The stained microslides were visualized and photographed using a Zeiss Axioskop 40 microscope equipped with an Axiocam camera.

mRNA expression of lineage-specific markers (osteocalcin, RunX2, PPARγ and adiponectin) in iMSC cultures was detected at the passage 26th using primers and amplification parameters listed in Table S1. Since iMSCs at later passages (after 30th) were poorly differentiated, no PCR for these passages was performed.

### 4.8. Senescence-Associated Beta-Galactosidase Staining

To evaluate the cell culture senescence, we performed the senescence-associated beta-galactosidase staining, according to the previously published protocol [34]. Briefly, cell cultures were fixed for 5–7 min in 2% glutaraldehyde solution and washed three times with 0.1 M PBS (pH 7.4) for 5 min each. Staining was performed in 2 mM MgCl_2_, 5 mM potassium ferrocyanide, 5 mM potassium ferricyanide, 0.01% sodium deoxycholate and 0.02% Nonidet P-40 solution containing 1 mg/mL 5-bromo-4-chloro-3-indolyl-beta-D-galactoside (X-Gal), at 37 °C overnight. The images were captured using a Nikon Eclipse Ti2 microscope equipped on a *DS-Fi3* camera (Nikon, Melville, NY, USA)**.**

### 4.9. Statistical Analysis

Statistical analysis was performed using the SigmaPlot11.0 program (Systat Software, Inc., Erkrath, Germany). Numerical data were evaluated for normality of distribution using the Kolmogorov–Smirnov criterion. Differences between experimental and control groups were analyzed using Student’s *t*-test or ANOVA (analysis of variance) for multiple pairwise comparison (Tukey test). Data are presented as mean ± standard deviation (MSC proliferation and hTERT activity) or mean ± standard error of the mean (hormone-induced signaling and differentiation). Differences between groups were considered significant at *p* < 0.05.

## 5. Conclusions

In this study, we obtained and characterized novel immortalized adipose tissue-derived MSC cultures suitable for studying MSC biology. Obtained iMSC cultures retained the properties of young MSCs, such as immunophenotype, proliferative activity, differentiation potential, hormone sensitivity and sensitization capacity, until at least 17–26 passages. Although not indefinitely long, the iMSC cultures provide the possibility for scaling and standardizing the research object (including the experiments involving genome-editing technologies). Meanwhile, the research potential of a primary MSC culture is rather restricted due to their prompt senescence by the 8–12 passages and prominent donor-specific variation. The relatively high proliferative potential of the obtained iMSC cultures combined with the retained MSC-specific physiological properties make these cultures a feasible model object for studying the mechanisms of MSC responses to hormones and neuromediators as well as other aspects of MSC biology.

## Data Availability

Data are available on request.

## Supplementary Materials

The following supporting information can be downloaded at: https://www.mdpi.com/article/10.3390/ijms25042421/s1.
