# Supplementary material for: Novel Immortalized Human Multipotent Mesenchymal Stromal Cell Line for Studying Hormonal Signaling
Source: Int J Mol Sci. 2024 Feb 19;25(4):2421. doi: 10.3390/ijms25042421 (PMC10889231; doi:10.3390/ijms25042421)
Supplement: Supplementary file 1 [file ijms-25-02421-s001.zip › ijms-2811845-supplementary.pdf]

Table S1. Primer sequences and amplification parameters used in the study.

Gene	Primer name	Nucleotide sequence (5′ -> 3′)	Annealing T, ⁰C	Amplicon length, bp
TERT	TERT_qPCR_f	ACCGTGGTTTCTGTGTGGTG	58	211
	TERT_qPCR_r	TCGCCTGAGGAGTAGAGGAA		
RPLP0	RPLP0_h_f	GCTGCTGCCCGTGCTGGTG	63	130
	RPLP0_h_r	TGGTGCCCCTGGAGATTTTAGTGG		
RunX2	RunX2_f	TCTTAGAACAAATTCTGCCCTTT	56	136
	RunX2_r	TGCTTTGGTCTTGAAATCACA		
OCN/ BGLAP	OCN_f	AGCAAAGGTGCAGCCTTTGT	58	62
	OCN_r	CGCCTGGGTCTCTTCACT		
PPARG	PPARg_f	TCAGGTTTGGGCGGATGC	59.5	147
	PPARg_r	TCAGCGGGAAGGACTTTATGTATG		
AdipoQ	AdipoQ_f	GACCAGGAAACCACGACTCA	59	199
	AdipoQ_r	TTTCACCGATGTCTCCCTTAGG		

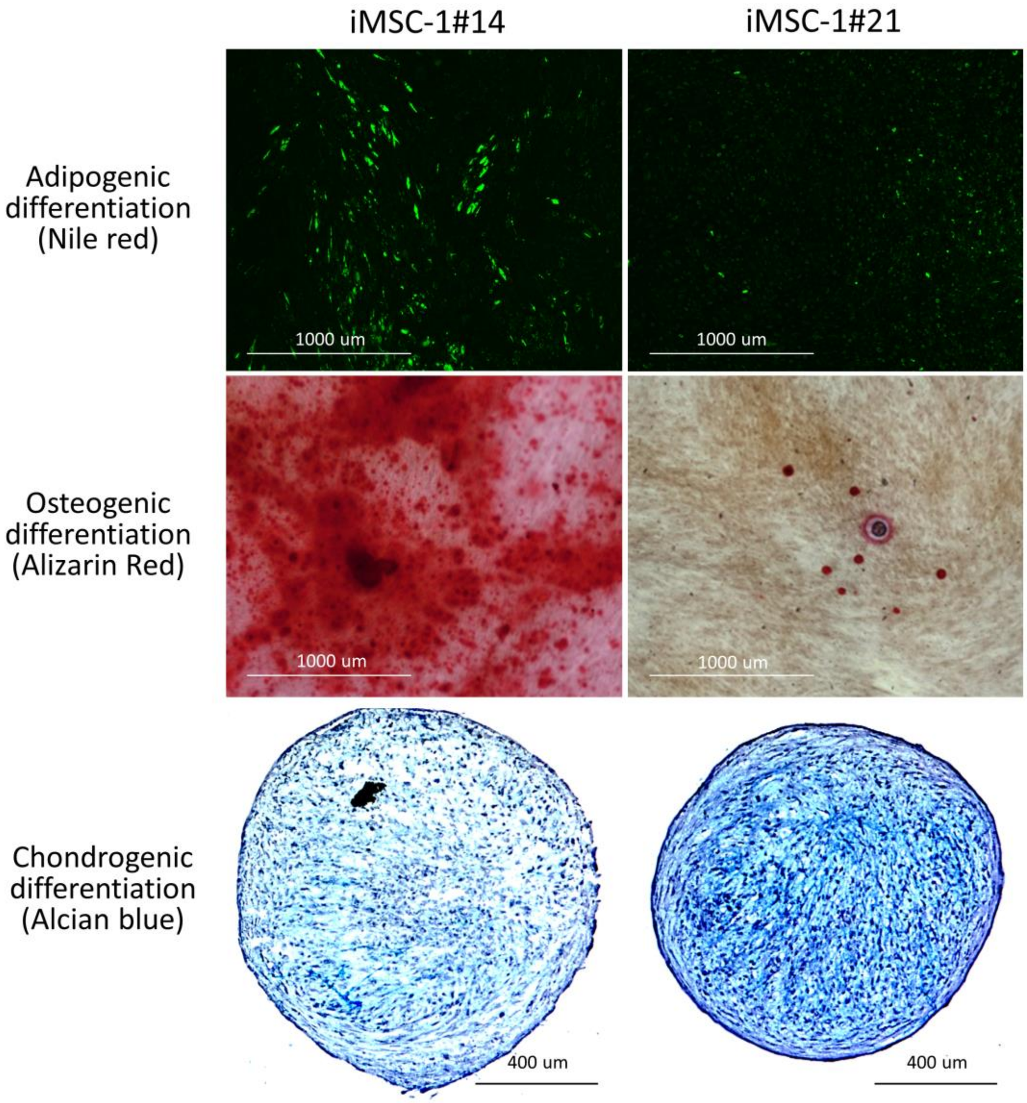

Figure S1. iMSC-1 differentiation potential at passages 14 and 21: MSCs cultured in appropriate differentiation medium for 2 weeks. Adipogenic differentiation was assessed by Nile red staining of accumulated lipids (green fluorescence), osteogenic differentiation - by Alizarin red staining of calcium deposits (red color) and chondrogenic differentiation - by Alcian blue staining of cartilage acidic glycosaminoglycans (light blue color).

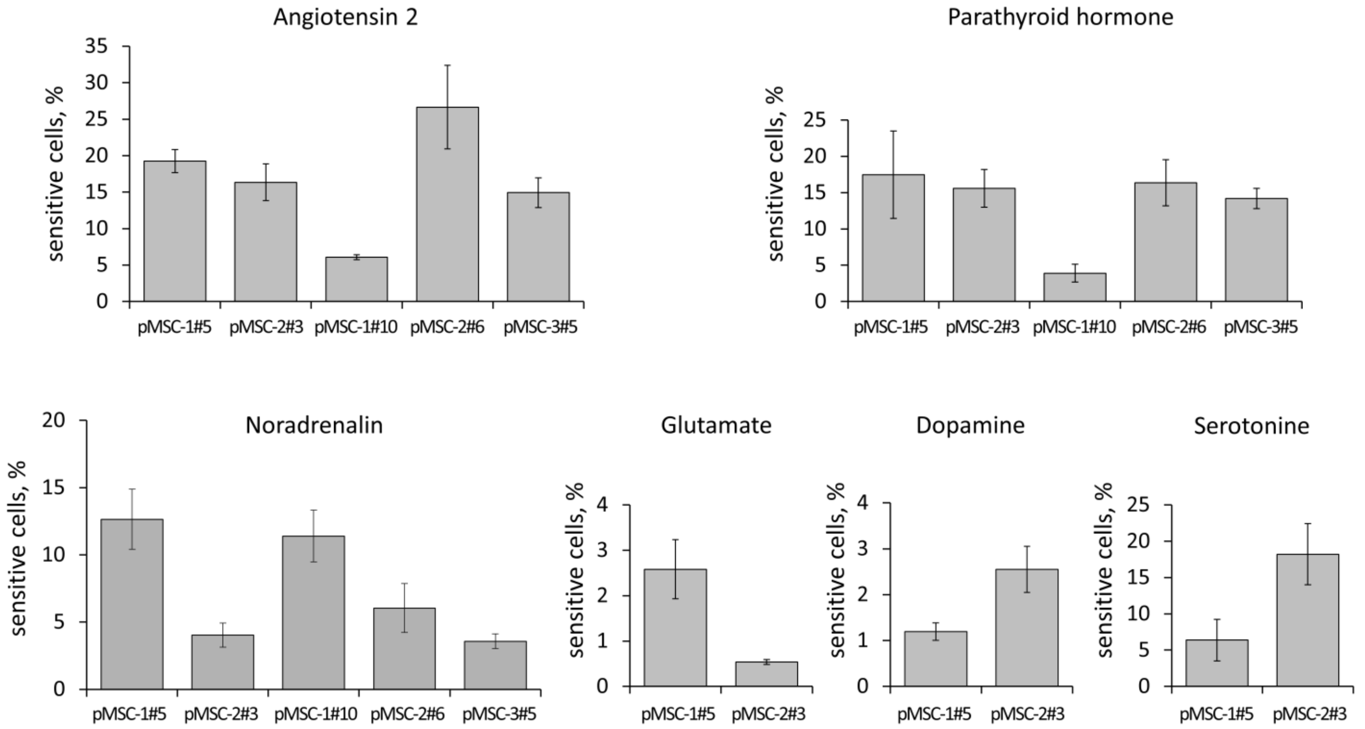

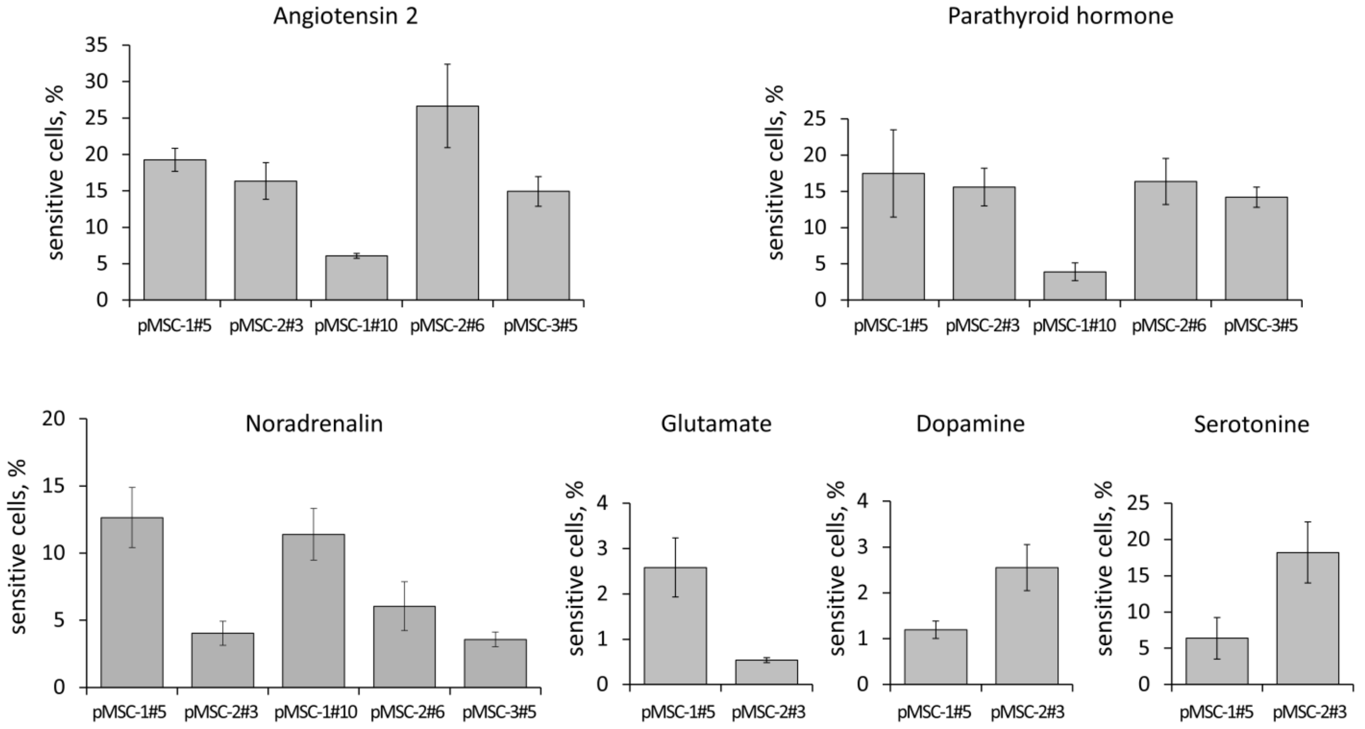

Figure S2. Hormonal sensitivity of primary MSC cultures (#1-3) at passages 3-10 (n ≥ 3).

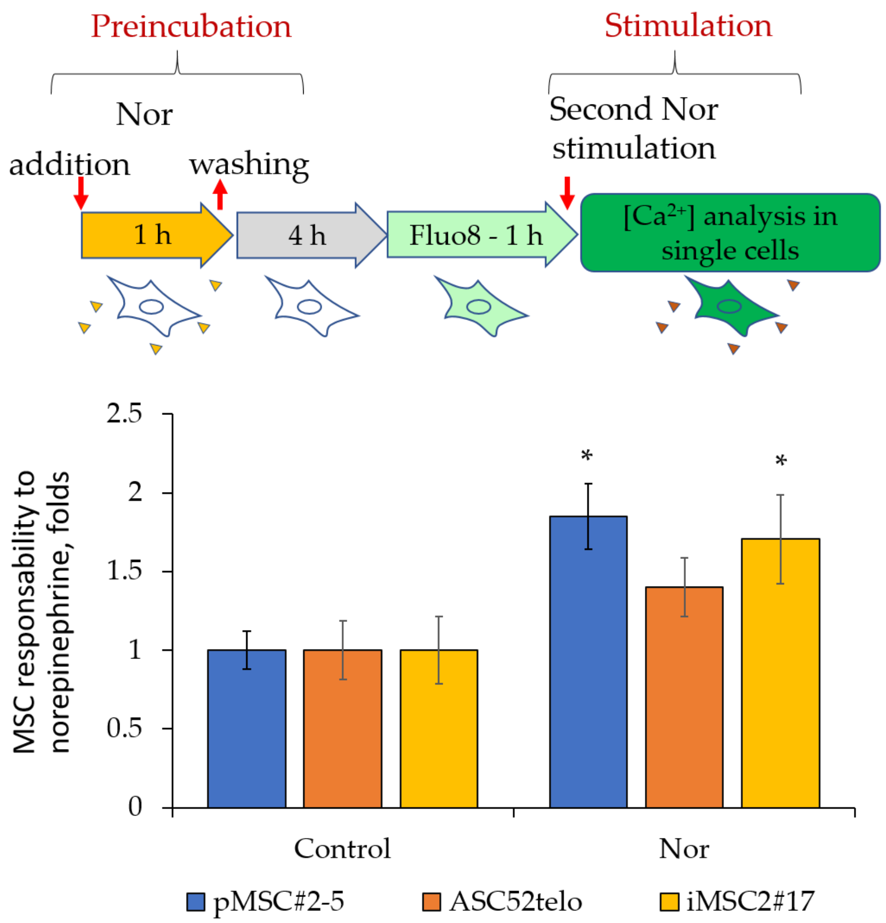

Figure S3. Hormonal sensitization of iMSC cell culture. * - $p < 0.05$ vs corresponding cell culture in the control group (non-pretreated with noradrenaline), $n \geq 6$.

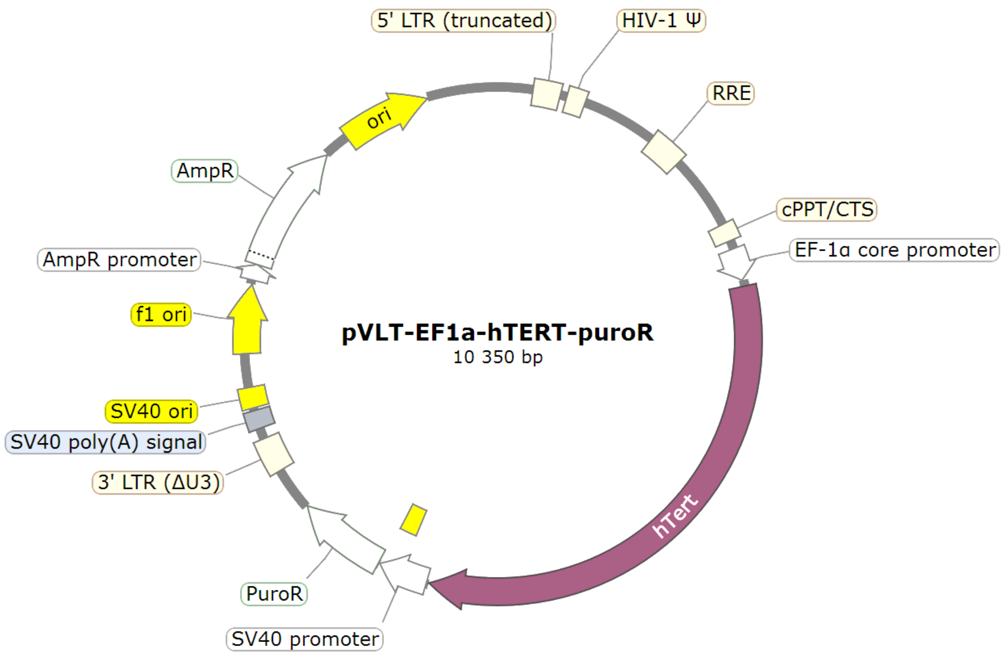

Figure S4. Map of the lentiviral transfer vector pVLT-EF1a-hTERT-puroR used for immortalization of MSC cell cultures.
